# Effects of sex and gender on the etiologies and presentation of select internalizing psychopathologies

**DOI:** 10.1038/s41398-024-02730-4

**Published:** 2024-02-02

**Authors:** Kritika Singh, Frank R. Wendt

**Affiliations:** 1 Division of Genetic Medicine, Department of Medicine, Vanderbilt University Medical Center, Nashville, TN USA; 2 Vanderbilt Genetics Institute, Vanderbilt University Medical Center, Nashville, TN USA; 3 Biostatistics Division, Dalla Lana School of Public Health, University of Toronto, Toronto, ON Canada; 4 Department of Anthropology, University of Toronto, Mississauga, ON Canada

**Keywords:** Psychiatric disorders, Genomics

## Abstract

The internalizing spectrum encompasses a subset of psychopathologies characterized by emotional liability, anhedonia, anxiousness, distress, and fear, and includes, among others, diagnoses of major depressive disorder (MDD), generalized anxiety disorder (GAD), and posttraumatic stress disorder (PTSD). In this review, we describe the vast body of work highlighting a role for sex and gender in the environment, symptom onset, genetic liability, and disorder progression and comorbidities of MDD, GAD, and PTSD. We also point the reader to different language used in diverse fields to describe sexual and gender minorities that may complicate the interpretation of emerging literature from the social sciences, psychiatric and psychological sciences, and genetics. Finally, we identify several gaps in knowledge that we hope serve as launch-points for expanding the scope of psychiatric studies beyond binarized sex-stratification. Despite being under-represented in genomics studies, placing emphasis on inclusion of sexual and gender diverse participants in these works will hopefully improve our understanding of disorder etiology using genetics as one tool to inform how biology (e.g., hormone concentration) and environmental variables (e.g., exposure to traumatic events) contribute to differences in symptom onset, pattern, and long-term trajectory.

## Introduction

The Hierarchical Taxonomy of Psychopathology endorses five spectra of psychopathology, including somatoform, internalizing, thought disorders, detachment, and externalizing (sometimes separated into disinhibited and antagonistic externalizing spectra) [1, 2]. The somatoform spectrum of psychopathologies causes individuals to experience physical ailments like pain, gastrointestinal upset, and altered cognition. Thought disorders manifest with symptoms of mania, dissociation, and reality distortion, and include diagnoses like schizophrenia and bipolar disorders. The detachment spectrum of psychopathologies comprises emotional detachment, anhedonia, and social withdrawal, and may include avoidant personality disorders and schizoid personality disorder. The externalizing spectrum is characterized by symptoms like rule breaking, aggression, poor impulse control, and inattention. Disorders on the externalizing spectrum include attention deficit hyperactivity disorder, conduct disorder, borderline personality disorder, and substance use disorders (SUDs).

This review covers the internalizing spectrum of psychopathologies which comprises symptoms of emotional liability, anhedonia, anxiousness, distress, and fear. Diagnoses belonging to the internalizing spectrum are vast and include major depressive disorder (MDD), agoraphobia, obsessive compulsive disorder, generalized anxiety disorder (GAD), posttraumatic stress disorder (PTSD), social phobias, and anorexia nervosa. We focus this review on three common internalizing disorders (MDD, GAD, and PTSD), their symptoms, and their comorbidities, as these traits have (i) numerous large genetic studies informing their underlying etiology, (ii) evidence of sex- and gender differences in their presentation and symptom patterns, and (iii) robust environmental factors associated with symptom onset, disorder progression, and comorbidity.

Various psychiatric conditions have shown differences based on biological sex (usually determined by the absence or presence of a Y-chromosome) and gender but many studies use these terms interchangeably. Sex and gender are multidimensional constructs, which exist on a spectrum. Even though dichotomized dimensions of sex and gender have been studied, there is very little research on how continuous dimensions of sex and gender relate and contribute to internalizing psychopathologies. We hope this article prompts warranted discussions related to going beyond the historical binary of genetic study design, terminology, and existing evidence supporting changes to future work in psychiatry.

## Definitions

The bulk of this review covers several layers of study supporting a role for sex and gender in the presentation and/or biology of internalizing disorders and related psychopathologies. The term “sex” in epidemiological and clinical studies is often determined through self-reported surveys and typically overlaps with one’s self-reported sexual or gender identity [3]. In genetic studies, “sex” is routinely dichotomized through a quality control phase of SNP-array preprocessing in which the presence or absence of Y-chromosomal loci is detected per individual. In some instances, the number of X- and Y-chromosomes also may be estimated from SNP-array data. The term “sex” as used in genome-wide association studies of psychiatric disorders refers to a binary classification of an individual into “male” (i.e., someone who carries one Y-chromosome) or “female” (i.e., someone who does not carry a Y-chromosome). In one common genetic data quality control pipeline, using PLINK for example, individuals are called female if their X-chromosome homozygosity estimate is <0.2 and as male if the estimate is >0.8. Transgender and nonbinary participants and study participants with different arrangements of sex chromosomes are relatively sparse in existing genomic studies. In the often-studied UK Biobank, for example, the prevalence of other combinations of sex chromosomes is 0.17% (213 participants with XXY and 143 participants with XYY sex-chromosomal arrangements) [4]. Despite sex being routinely dichotomized by the methods described above, we must appreciate that this practice merely reflects two frequent sex-chromosomal arrangements and undermines the multidimensional nature of sex [5]. This necessarily reduces the generalizability of GWAS findings to individuals with other chromosomal arrangements, even if relatively rare in commonly studied biobanks.

Important and dedicated attention is now being given to internalizing diagnoses and symptom patterns among sexual and gender minorities. Unlike the common approach to dichotomizing sex, “gender” is readily understood on a spectrum. Gender and sexual minorities include, but are not limited to asexual, bisexual, gay, intersex, lesbian, nonconforming, queer, transgender, and Two-spirit. Sexual minorities are not typically removed from GWAS unless they are also a gender minority, but selection of sexual minorities to include in a GWAS may reduce generalizability [6]. Among two commonly studied biobanks, the prevalence of sexual and gender minority identities ranged drastically due, in part, to cohort ascertainment and study inclusion criteria. For example, in the Million Veteran Program (MVP), historical practices for enrollment of military personnel contributed to the presence of relatively few transgender and non-heterosexual persons [7]. In studies that intentionally recruited minoritized veterans, sample sizes were similarly sparse [8]. Conversely, using the UK Biobank and 23andMe, one study stratified participants by their endorsement of “any” versus “no” same-sex partnering behavior [9] (range in prevalence from 3.5% to 18%). In doing so, the authors included primarily bisexual, heterosexual, homosexual participants which may lead to widespread public confusion about genetics and sexual behavior [6]. Participants may be excluded from genetic studies if, among other reasons, (i) their sex-chromosomal arrangement is inconsistent with a pattern conventionally classified as “male” or “female” via the presence of one Y- and one X-chromosome (“male”) or the presence of two X- and zero Y-chromosomes (“female”) or (ii) their self-identified gender or sex are inconsistent with their sex-chromosomal pattern as determined by SNP-array. In practice, these “sex-check” steps of genotyping array quality control may be considered evidence of contamination of the samples by a laboratory technician, sample collector, and/or intimate partner (if, for example, buccal/saliva samples are the source of the data) [10, 11]. Furthermore, the structure of participant recruitment surveys may contribute to underrepresentation of sexual and gender minorities in publicly available data and supports study designs that explicitly include diverse identities rather than prospective investigation of available data [12]. By including more diverse options in recruitment questionnaires, discordant information between the sex-check quality control phase and participant identity can be contextualized and included in study reports rather than omitted without discussion.

Taken together, this review makes every attempt to highlight epidemiological and clinical studies that pay necessary attention to sexual and gender minority health. Our discussion uses various terms such as “men,” “nonbinary persons,” “transgender persons,” “women” to describe study participants with language consistent with the cited literature. Among the genetic studies presented, there are investigations that use gender-inclusive language but, upon reviewing their methods, actually report data for a cohort separated by the presence of XX or XY sex-chromosomal arrangements. We aim to present source material using the authors’ original language and study designs while highlighting where use of language is inconsistent with methodology. In these scenarios, we make no claims regarding the gender and sexual minority inclusivity of the study. Rather, we highlight areas where the science has evolved and where this topic could be improved or, at the very least, openly discussed in the future literature with a goal of improving representation and care.

## Symptom presentation

### Major depressive disorder

The Diagnostics and Statistical Manual of Mental Disorders, fifth edition (DSM-5) defines MDD as a serious mood disorder with persistent feelings of sadness and hopelessness and loss of interest in activities once enjoyed (also called anhedonia). The DSM-5 criteria for MDD diagnosis include five or more symptoms reported in the last two-week period with at least one of those symptoms being (i) depressed mood or (ii) anhedonia. These symptoms must cause significant distress to the individual or impair their social, occupational, or other important areas of functioning. Across several depression scales, women tend to report a higher burden of depressive symptoms even though men report a greater number of depressive episodes [13]. Among individual depressive symptoms, adult women were more likely to report changes in appetite, frequent tears, loss of interest, and thoughts of death [14]. A study of Canadian older adults reinforced these the difference in broad depression between men and women but did not pinpoint specific symptoms differing between these two genders [14]. Early-life symptom trajectories support differences between boys and girls. Early-life trajectories for girls consistently showed increasing symptom severity as they age while boys have inconsistent age-based symptom trajectories [15]. Depression is also highly comorbid with various other conditions, particularly anxiety [16] and cardiovascular disease [17]. It is interesting to note that even though the population level prevalence of depression is higher in women relative to men, cardiovascular diseases are significantly more abundant in men [18]. Exploring this relationship has uncovered a potentially mediating effect of inflammatory biology that may be especially relevant in women [19–21].

### Generalized anxiety disorder

The American Psychiatric Association defines anxiety as a normal response to stress which can even be beneficial in some situations, such as increasing attention and focus on a test or work task. However, anxiety disorders differ from *temporary* feelings of anxiousness or nervousness with more *intense* feelings of fear or anxiety. The DSM-5 specifically describes anxiety disorders as excessive worry and apprehensive expectations, occurring more days than not for at least 6 months. Anxiety and worry are associated with three or more of the following six symptoms with at least some symptoms present for more days than not for the past 6 months: (a) restlessness or feeling keyed up or on edge; (b) easily fatigued; (c) difficulty concentrating or mind going blank; (d) irritability; (e) muscle tension; (f) sleep disturbance such as difficulty falling or staying asleep, or restless and unsatisfying sleep. Anxiety disorders are the most common mental health concern globally [22], and it’s estimated that 19.1% of US adults have an anxiety disorder [23]. This estimate approached 40% of the adult population at the height of the COVID-19 pandemic [24]. Between 5–12% of children under 18 experience anxiety issues each year, and most people develop symptoms before age 21 [25]. Women are more likely than men to have anxiety disorders [26]. By age 6, girls were already twice as likely as boys to have experienced an anxiety disorder [26]. There are six different types of anxiety disorders - generalized anxiety disorder, social anxiety disorder, panic disorder, phobias, separation anxiety disorder and substance or medication-induced anxiety disorder. Women self-rate their anxiety symptoms worse than men [27] and diagnostic instruments support these reports, with women scoring 12.3% higher than men for somatization, interpersonal sensitivity and panic [28]. Women were more likely to suffer from comorbid depression and bulimia nervosa, and less likely to have a comorbid SUD [27]. Most large studies focus on GAD which has an age of onset in early adulthood with symptoms persisting into later life [26]. Men with GAD had higher rates of comorbid SUDs, nicotine dependence, and antisocial personality disorder [26]. Women with GAD had higher rates of comorbid mood disorders (except bipolar disorder) and other anxiety disorders (except social anxiety disorder) [29]. Despite women having greater odds of an anxiety disorder diagnosis, there has been no reported difference in social anxiety disorder [26]. The lifetime and 12-month man:woman prevalence of any anxiety disorder were 1:1.7 and 1:1.79, respectively [26]. Although there were no significant differences between men and women in age of onset within various racial categories, there was a significant interaction between gender and race such that the age of onset for social anxiety disorder was lower (mean age = 11.4 years) among European American men than among African American women (mean age = 13.8 years) [26].

### Posttraumatic stress disorder

PTSD is a psychiatric disorder that may arise in response to a severe traumatic event. The DSM-5 advises diagnosis of PTSD based on four symptom clusters (re-experiencing, avoidance, negative alterations in cognition/mood, and hyperarousal/hypervigilance). Some of the material in this review, including genetic investigations, rely on prior PTSD definitions including the DSM-IV, which recognizes the re-experiencing, avoidance, and hyperarousal/hypervigilance symptom domains [30, 31]. The reported lifetime prevalence of PTSD is about 10–12% in women and 5–6% in men [32].

PTSD is unique among psychiatric disorders in its requirement for an index trauma. Men and women experience different types of trauma with women reporting more frequent exposure to sexual trauma than men, and at a younger age [20]. Conversely, men are more often exposed to physically violent trauma such as combat or exposure to war [33]. These differences even extend to specific historical events; for example, (i) internally displaced Iraqi women reported more somatic (*p* < 0.001) and depressive/anxious (*p* < 0.001) symptoms than men [21] and (ii) following the 2011 bombing in Olso, Norway, women employees of Norwegian ministries had greater symptoms of re-experiencing the event and elevated startle response compared to men [34]. Though exposure to different traumas is a major driver of PTSD symptom differences between men and women, gender differences in symptom severity persist even after controlling for trauma differences. Studies that take this approach may inform relevant processes related to increased susceptibility in women [35, 36].

The PTSD symptom clusters recognized by DSM-5 are re-experiencing (sudden and unwanted traumatic memories that intrude into or even seem to replace what’s happening now), behavioral avoidance (avoiding reminders on a trauma such as like places, people, sounds or smells), hyperarousal (the fight-or-flight or fight-flight-or-freeze response in response to a perceived harmful event, attack, or threat to survival), and negative alterations in cognition and/or mood following (e.g., persistent negative beliefs, emotional state, self-image). These clusters represent unique underlying biology to a certain degree [37] and cluster scores are consistently higher in women [38]. It has also been indicated that women expressed more distress than men across almost all the symptoms on the PTSD Checklist except for hypervigilance. Compared to men, women experienced more re-experiencing symptoms and were more likely to meet criteria for current PTSD, they also were more likely to report sexual trauma as their index trauma [39]. Elevated symptoms in women may be attributed, in part, to hypothalamus-pituitary-adrenal (HPA) axis activity. In a longitudinal study of civilian trauma and PTSD, Shalev et al. performed a gender-by-diagnosis analysis and showed that plasma concentration of adrenocorticotropin associated with greater PTSD symptoms at multiple time points in women only [40].

PTSD has a complex network of comorbidities that associate with sex and/or gender of the person with the diagnosis. In a large study of U.S. Sailors and Marines, women were more likely than men to have PTSD with comorbid adjustment disorder, major depressive disorder, and generalized anxiety disorder or other anxiety disorders, with the largest effect for eating disorder (OR = 12.6). Conversely, women were less likely to experience comorbid SUDs, sleep disturbances and disorders, and traumatic brain injury (OR = 0.17) [41]. Many of these effects persist after rigorous covariation for other co-occurring conditions like suicide attempt. Many of these gender differences can at least partially be explained by type of index trauma reported [42].

### Transdiagnostic psychopathologies

Men and women exhibit relatively large differences in diagnostic prevalence and symptom manifestation of various psychiatric and mental health disorders. We talk in detail about a few of them above, but various transdiagnostic metrics of these disorders also show a great extent of differences among sex-chromosome stratified cohorts that mirror gender differences. These differences can be attributed to various causes including underlying biology and social structure. For example, women reported higher neuroticism than men [43].

Personality may play a large role in one’s presentation of internalizing symptoms. Neuroticism describes the tendency to experience negative emotion and related processes in response to perceived threat and punishment; these include anxiety, depression, anger, self-consciousness, and emotional lability. Women have been found to score higher than men on neuroticism as measured at the Big Five trait level, as well as on most facets of neuroticism included in the NEO Personality Inventory-Revised [44]. The one facet of neuroticism in which women do not always exhibit higher scores than men is anger, or angry hostility [44]. Mean and covariance structure models testing gender differences at the level of latent traits revealed higher levels of neuroticism (*d* = 0.52) and agreeableness (*d* = 0.35) in older women compared to older men [44]. While agreeableness did not play a major role in this model, it did significantly influence the understanding of gender differences within groups. Women were found more agreeable in participants with MDD and controls, but those from the control group were more open and conscientiousness than control men [45]. Women also score somewhat higher than men on some facets of conscientiousness, such as order, dutifulness, and self-discipline [44, 46]. These differences are not consistent across cultures and no significant gender difference has been found in conscientiousness at the Big Five trait level [44]. Lastly, women tend to score higher than men on warmth, gregariousness, and positive emotions, whereas men score higher than women on assertiveness and excitement seeking [44, 46]. All five-factor model (i.e., Big Five) traits have been associated with PTSD symptoms but neuroticism showed the strongest relationship [47]. Further analyses considering gender in their model revealed a stronger effect for men and an effect in women mediated by peritraumatic emotions and dissociation [48].

Suicidal thoughts and behaviors are strongly associated with mental health outcomes but have considerable overlap with internalizing diagnoses such that some aspects of the PHQ-9, for example, may be used to derive a quantitative assessment of severity of suicidal thought [49–52]. Women with a PTSD diagnosis were 6.74-times more likely than women without PTSD to attempt suicide after adjustment for many socioeconomic variables. Among men, the difference was 3.96-times [53]. Common risk factors of suicidal behaviors shared across gender identity are previous mental health diagnosis and childhood sexual abuse [54, 55]. Risk factors for suicide attempts specific to women include eating disorders, PTSD, bipolar disorder, being a victim of dating violence, depressive symptoms, interpersonal problems and previous abortion [56]. Risk factors for suicide attempt specific to men include disruptive behavior/conduct problems, hopelessness, parental separation/divorce, friend’s suicidal behavior, and access to means of taking one’s own life [56]. It has been reported that men die by suicide more frequently than women, but women more often make suicide attempts [55, 56]. Based on 2018 data from the United States, the age-adjusted suicide rate for men (22.4 per 100,000) was 3.67 times larger than for women (6.1 per 100,000) [57].

Since psychosocial problems are more common among women than men, the impact of sleep and associated disorders affects women much more than men. The prevalence of depression is higher in women and was linked to length of sleep during early adolescence suggesting that sleep may serve as one tool for mitigating depression symptoms over time [58]. Furthermore, among adult caregivers in a family, women who serve as the primary caregiver reported greater stress, depression symptoms, and sleep disturbances than men who serve as caregivers [59]. Normal sleep among women is impacted by hormonal effects during menses, pregnancy/lactation, perimenopause, menopause, and post-menopause and often leads to sleep disturbances and changes in mood during these periods [60]. For example, one-third of women report cramps, bloating, and headaches as reasons for disrupted sleep during the premenstrual phase or during menses [59].

### Sexual and gender diversity

Sexual and gender minority populations now have deep scientific support for the nuanced relationship between lived experience and mental health outcomes related to internalizing psychopathologies. In one study, sexual and gender minorities reported substantially higher depressive symptoms and more frequent suicidal thoughts and behaviors (odds ratio up to 5.44) [61]. This same study also highlighted pronounced differences in internalizing symptoms among different populations of self-identified race and ethnicity. Asian/Pacific Islander participants in this study were less likely to report suicide attempt (odds ratio = 0.45) while LatinX participants were more likely to report a suicide attempt (odds ratio = 1.50) [61]. Though appreciable sample sizes were reported, there was no notable interaction between race/ethnicity and sexual or gender minority identities with respect to odds of internalizing symptoms [61]. However, there was strong support for greater depressive symptoms (as measured by the PHQ-9) among cisgender women, transgender men, transgender women, nonbinary respondents, bisexual, queer, and pansexual minorities relative to cisgender men in a large Canadian sample [62]. In this study, the gender minority with the largest PHQ-9 was nonbinary respondents (beta = 3.70) and the sexual minority with the largest PHQ-9 was pansexual respondents (beta = 1.90). This effect appears to also capture socioeconomic and race/ethnicity effects on depressive symptoms which is consistent with documented effects of socioeconomic position on depression [63].

In addition to a larger burden of depressive symptoms among sexual and gender minorities, these groups report greater frequency of adverse life events. Relative to heterosexuals with no same-sex attraction or past partners, lesbians, gay men, bisexuals, and heterosexuals who reported any same-sex sexual partners over their lifetime had greater risk of childhood maltreatment, interpersonal violence, trauma to a close friend or relative, and unexpected death of someone close to them [64]. Not unique to sexual and gender minorities is the complexity and heterogeneity of index trauma and PTSD symptomology. For example, sexual and gender minority participants with a PTSD diagnosis reported significantly more unwanted sexual contact, sexual and physical assaults, and other severe suffering experiences. The same participants reported greater eating disorders, depressive symptoms, and anxiety symptoms than controls without PTSD (but are also sexual and gender minorities) or other PTSD cases who do not identify as a sexual or gender minority [65]. Some evidence suggests that specific traumatic events, such a child abuse, may account for one-third to one-half of the PTSD disparities among sexual and gender minorities [64]. One important resource for studies of PTSD in sexual and gender minority groups is large healthcare systems like the United States Veterans Health Administration Hospital System. While sexual and gender minority military veterans make up a small portion of the system, such large cohorts of homogeneously ascertained personnel with consistent healthcare can be one avenue to overcome heterogeneity in recruited cohorts. Among almost 10,000 transgender veterans, the incidence of PTSD diagnosis was up to 1.8 times higher than cisgender veterans matched on age group at first healthcare visit, sex assigned at birth, and year of first visit [66]. Furthermore, descriptive data suggest that the prevalence of depression, schizophrenia, bipolar disorder, alcohol and non-alcohol SUDs, current/former smoking status, and military sexual trauma was also elevated among transgender veterans [66].

A major risk factor for internalizing symptoms and subsequent diagnoses among sexual and gender minorities is discrimination. Several self-report assessment tools exist to evaluate discrimination in the form of everyday stressors (e.g., frequency of discrimination based on orientation and/or race/ethnicity), major experiences (e.g., being fired from a job, being denied a job, being denied housing because of orientation and/or race/ethnicity), and chronic workplace experiences (e.g., frequency of interpersonal experiences at work). Recently reviewed by Livingston et al. [67] evaluation of these assessments among sexual and gender minority populations reveal that work/school discrimination and harassment predicted between 19-37.5% of variance in suicidal ideation [67]. It also appears that participants who report sexual and gender based discrimination as an index trauma exhibit unique patterns of anxiety and stress symptoms. Among adults who experienced trauma, those who note discrimination based on sexual and/or gender orientation had higher attachment anxiety, attachment avoidance, emotion dysregulation, dissociative symptoms, and greater incidence of PTSD diagnosis [68]. Taken together, the many forms of discrimination play a large role in reports of internalizing psychopathology among sexual and gender minorities. Efforts to mitigate these events may have large positive effects on mental health in schools and the workplace, especially for minorities in those spaces.

## Genetic investigations

Genome-wide association studies (GWAS) perform statistical tests for the relationship between internalizing traits and diagnoses and the dose of an allele at a given position of the genome [69–84]. Many large-scale efforts exist to perform GWAS of psychiatric conditions. The Psychiatric Genomics Consortium (PGC) and MVP lead these efforts and often collaborate with scientists from the direct-to-consumer genetic testing company 23andMe, Inc. and biotechnology companies like Regeneron Pharmaceuticals, Inc. Relatively few GWAS are performed in cohorts stratified by chromosomal sex patterns due to nearly halving the statistical power of the study. We introduce below various studies that primarily stratify participants based on XX and XY sex-chromosomal arrangements.

In a sex-stratified GWAS of depression [85], one locus associated with MDD in males but could not be replicated in an external cohort and was not tested for male-specificity. Furthermore, five variants called from whole exome sequences of participants with an MDD diagnosis were significantly different between males and females [86]. These mapped to phosphodiesterase 4A (*PDE4A*), ferredoxin 1 like (*FDX1L*), and myosin-XVB (*MYO15B*). Participants that carried these variants showed younger age of depression onset, greater incidence of suicide attempt, greater odds of family history of depression, and greater incidence of recurrent depression [86]. While internalizing diagnoses have received little sex-stratified attention in the literature, some transdiagnostic risk factors have garnered attention via sex-stratified investigations. Neuroticism is one transdiagnostic feature of internalizing disorders with several sex-specific and sex-stratified studies [87, 88]. These studies report no differences in sex-stratified SNP-based heritability for neuroticism. However, in silico follow-up studies of sex-stratified neuroticism GWAS support some unique polygenic features in XX and XY carrying participants that could be relevant for therapeutic targeting among these participants. These include female-specific (XX sex-chromosomal pattern) red blood cell features and plasma calcium concentration [88].

**Fig. 1:**
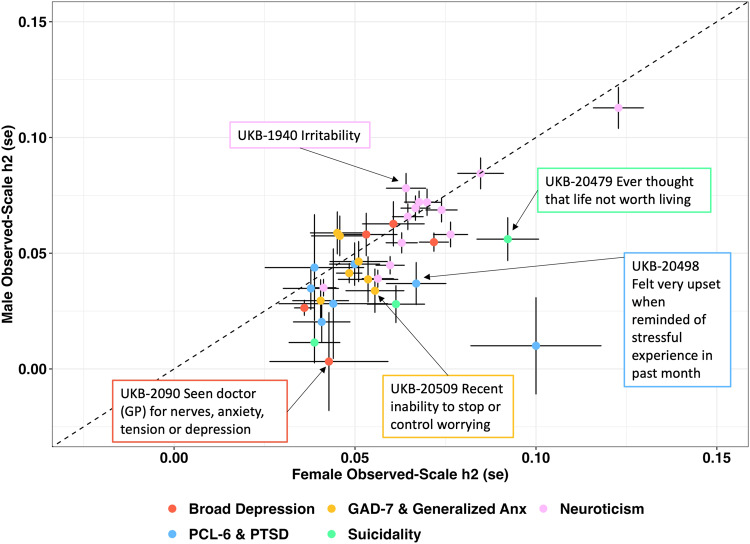
SNP-based heritability (*h*^2^) estimates for internalizing traits and transdiagnostic features of the internalizing spectrum from publicly available resources including the UK Biobank (UKB) and the Psychiatric Genomics Consortium. Observed-scale SNP-based *h*^2^ was estimated using Linkage Disequilibrium Score Regression and the 1000 Genomes Project European ancestry linkage disequilibrium reference panel. Traits are colored by primary trait and/or measure of symptom severity. Each data point represents trait *h*^2^ with crosshairs reflecting the standard error (se) in females (horizontal crosshairs) and males (vertical crosshairs). For each domain, the largest significant difference between males and females is labeled. Significance (*p* < 0.05) was determined using two-sided Z-tests. PCL-6 = PTSD Checklist 6-item questionnaire, GAD-7 = generalized anxiety disorder 7-item questionnaire, UKB UK Biobank, GP general practitioner.

Figure 1 compares the observed-scale SNP-based heritability estimates of several internalizing disorders and transdiagnostic features of the internalizing spectrum from the UK Biobank (http://www.nealelab.is/uk-biobank) and the PGC (https://pgc.unc.edu/for-researchers/download-results/). Observed-scale SNP-based heritability estimates were calculated using Linkage Disequilibrium Score Regression [68] and the 1000 Genomes Project European ancestry linkage disequilibrium reference panel. Several traits were significantly more heritable in females than males. These include most suicidal thoughts and behaviors, features of broad depressive symptoms, and the PTSD symptoms “upset feelings” and “avoidance”. Conversely, the neuroticism indicator trait “irritability” appears to have a slightly higher heritability among UKB males. Though heritability differences exist when comparing males and females, it is worth noting that in some contexts, heritability may be inflated in one sex due to ascertainment homogeneity [82]. For example, Huckins et al. reported an association between SNRNP35 and PTSD that was unique to military participants [89]. However, in the Million Veteran Program, PTSD genetic architecture was no different between combat exposed and unexposed veterans. Therefore, the effects reported by Huckins et al. [89] may reflect a broader unitary environment or matrix of shared environments instead of enrichment of a single large-effect shared index trauma. In combination, these observations warrant additional dedicated study of genetic factors associated with disorder symptoms in a sex- and trauma-stratified manner to better understand their unique etiologies and patterns of co-occurring disorders, symptoms, or environments. For example, Wendt et al. performed a sex-stratified genome-wide gene-by-environment interaction study of traumatic experiences, posttraumatic stress, and suicidal thoughts and behaviors. They detected unique patterns of environmental profiles and posttraumatic stress symptoms in males and females that could shape how genetic information contributes to suicidal thoughts and behavior [52].

Due, in part, to known differences in phenotype and trauma exposure, the PGC-PTSD working group appreciated early on the necessity of studying genetic factors associated with PTSD in stratified cohorts. PGC-PTSD Freeze 1 showed that PTSD in females had a SNP-based heritability significantly different from zero while males had no detectable genetic signal [69]. This same article supported polygenic overlap with PTSD and other adult psychiatric diagnoses like schizophrenia and bipolar disorder, but the statistical power of psychiatric GWAS at the time limited the scope of this comparison. In 2019, the PGC reported a second series of stratified genetic analyses of PTSD [78]. Despite the report of their findings using sex-chromosome stratified and gender stratified language interchangeably, they again showed that female SNP-based heritability was significant (10%, *P* = 8.03 × 10^−11^, Fig. 1), while male SNP-based heritability was not significantly different from zero (1%, *P* = 0.63, Fig. 1) [57]. Though not designed in a sex-stratified manner, the abundance of United States military service members who identify as men leads to a nearly male-specific GWAS of PTSD from the MVP after performing quality control to remove participants whose gender and sex-chromosome patterns are inconsistent with one another [82]. In this study, the SNP-based heritability of PTSD was significantly different from zero but a key finding from this study was the higher SNP-based heritability of a quantitative measure of posttraumatic stress symptom severity (8–10%) rather than binarized case-control (5–7%) definition. The authors also report greater power to inform PTSD biology from in silico analysis of GWAS data [82].

We have highlighted some large efforts to investigate genetic factors contributing to internalizing traits and disorders, most of which stratify their cohort using XY-chromosomal “males” and XX-chromosomal “females.” However, data stratified in this way are not the only way to uncover how sex and, in some cases, gender play a role in psychopathology. Again, coming out of the MVP, Levey et al. reported genome-wide significant loci associated with the GAD 2-item questionnaire, including a locus that positionally mapped to the estrogen receptor ESR1 [73]. Several studies support this finding with estrogen and estrogen receptor signaling being implicated in neuroprotection, neuroplasticity, and anti-inflammation. Because changes in hormone level disruptions are often reported among sexual and gender minorities relative to cis-gendered heterosexual study participants [90], this finding demonstrates one possible opportunity to learn about genetic effects on hormone receptor concentration and/or binding efficiency, and mental health among these communities. Though proposed by numerous small empirical studies, larger meta-analyses support the need for further investigation of hormone concentration differences across sex and gender minorities [91].

## Transcriptomic investigations

Transcriptomic analyses detect changes in gene expression associated with a given trait. The prefrontal cortex is one region of the brain most consistently impaired in internalizing disorders like major depression and posttraumatic stress disorders [92, 93]. Not only do brains of participants with a MDD diagnosis show altered prefrontal cortex transcriptional patterns relative to participants without MDD, but among participants with MDD there are stark differences between male and female transcriptional patterns [94]. For example, the female-specific hub gene *DUSP6* (encoding Dual Specificity Phosphatase 6) increases ERK signaling and pyramidal neuron excitability. This locus is routinely implicated in other psychiatric disorders, metabolic traits, and musculoskeletal features by GWAS. Similar male-specific observations have been made for the *EMX1* (encodes Empty Spiracles Homeobox 1) [94]. However, this locus has not been associated with any other trait at genome-wide significance (https://atlas.ctglab.nl/PheWAS). In addition to these hub genes serving as central mediators of transcriptional change, several gene sets exist that contribute to brain functional connectivity differences between males and females with depression [95].

With respect to PTSD, transcriptional changes may arise as (i) a consequence of traumatic experiences, (ii) as a possible cause of PTSD diagnosis, and/or (iii) as a consequence of PTSD symptoms. Similar to GWAS, untangling the heterogeneity of traumatic experiences reported by PTSD cases can be rather difficult. Sex-differences in brain region gene expression are pronounced [96] and gene expression differences between PTSD cases and controls are present [97]. One transcriptomic study of 52 PTSD cases reported several sex-specific genes localized to the prefrontal cortex [97]. In females, differentially expressed genes highlight pathophysiological importance of the orbitofrontal cortex and the subgenual prefrontal cortex regions while no such findings were identified for males. Furthermore, differential expression results only modestly overlapped between males and females, reinforcing some degree of specificity [97].

There remains a paucity of large sample size investigations of gene expression in internalizing disorders. In humans, the availability of brain tissue is limited to postmortem investigation and can be difficult to obtain and control for various confounders. There also is compelling evidence that postmortem data commonly used in brain-based transcriptomic studies poorly reflects gene expression in living tissue [98]. This point will be critical to consider in future study designs as access to such tissue may pose ethical and logistical challenges. In model organisms, studies of disorder symptoms can be informative of disorder biology in the context of preserved behavioral and neurobiology responses to stimuli as they are more easily measured by organism movement, ability to complete or learn a task, and social behavior. However, heterogeneity among external stimuli that trigger internalizing symptoms is highly relevant in humans (e.g., with PTSD index traumas) and may not be modeled effectively be a single model organism [99]. Finally, public gene expression data from resources like Gene-Tissue Expression (GTEx [100]) are suitably powered to generate sex-stratified weights to be used in transcriptome-wide association studies (TWAS). We anticipate stratified TWAS and GWAS to be a highlight of future research on internalizing diagnoses.

## Conclusions and future directions

We present a broad summary of the current state of sex-specific and sex-stratified genetic and transcriptomic studies of internalizing disorders and their transdiagnostic correlates. We articulate important differences in how sex and gender are used in the field, can be defined by researchers attempting to learn about internalizing spectrum disorders among sexual and gender minority communities, and attempt to stratify our summaries of this knowledge in a manner appropriate for how referenced authors describe their study cohorts. For example, in genetic studies, sex and gender have occasionally been used interchangeably to describe a cohort stratified solely on sex-chromosome patterns. Though some progress has been made to use genetic data to close the sex and gender gap in our understanding of internalizing psychopathologies, there are many areas requiring immediate attention to round-out the body of literature presented here.

First is explicitly considering sex and/or gender in biobank-level studies. From a GWAS perspective, many traits are suitably polygenic and suitably ascertained in large biobanks to justify stratifying the cohort into males and females for discovery of genetic effects unique to groups of people with XX or XY sex-chromosomal patterns. This is especially beneficial in the mental health research space due to large differences in diagnosis and presentation of disorder symptoms. For researchers concerned about splitting their cohort nearly in half (e.g., in the UK Biobank where nearly 55% of participants are female), other options may be more appropriate but should be used cautiously considering the research question at hand. The Structured Linear Mixed Model deployed by StructLMM is one way to model gene-by-sex interactions across the genome without loss of statistical power in the presence of many environments [50, 52, 101]. Depending on the research question, StructLMM may be performed considering sex and/or gender identity as one environment that interacts with genetic risk for disorder or behavior [101]. From an epidemiology perspective, if data are available for participants’ sexual and/or gender identities, these descriptive statistics should be reported along with age and other relevant parameters for the study even if the sample sizes are too small for formal inclusion in the primary model(s).

Our second recommendation draws on trait distribution. Many biobanks record internalizing symptoms on an ordinal scale but fit association models using linear assumptions. When phenotypic distribution in unbalanced (i.e., when extreme internalizing symptoms are scantly observed in a biobank), linear models of ordinal traits cannot control type-I error for relatively rare variants. Combining inflated type-I error rate with reduced statistical power described above, stratified study designs of ordinal traits may result in all false positives. One approach to enabling more rigorous stratified genetic studies of internalizing traits is application of a proportional odds logistic mixed model [102, 103]. Already applied to hundreds of traits during its development [102], this presents one potentially fruitful path forward for mental health GWAS that may permit the inclusion of more cohort strata in genetic association studies by improving statistical power through appropriate modeling of the ordinal distribution.

Whether the study is sex-stratified or not, very few genetic investigations of internalizing traits consider the X-chromosome. The X-chromosome is often overlooked because of the unique statistical approaches required to account for different X-chromosome dosage between males and females [104–106]. A few studies tackle the X-chromosome either in isolation or with the autosomes [88, 107] but this region of the genome remains grossly under investigated in internalizing disorder GWAS.

Finally, alluded to in the prior section is the lack of investigating sex-stratified gene expression effects for internalizing traits in humans. GTEx-v8 [100] contains 838 individuals with genotype and expression quantitative trait locus information. The sample is approximately 33% female and represents an opportunity for future TWAS to estimate sex-stratified gene expression weights for various applications of genetically-predicted gene expression.

Genetic studies of internalizing disorders, symptoms, and related behaviors have reached, or are approaching, their inflection points such that gene discovery is likely to expand in the next several years. Along with this inflection is the opportunity to expand our understanding of what specific and shared mechanisms influence internalizing traits in different strata of the full cohort. We have briefly described where the field stands and proposed several avenues forward to garner impactful findings regarding the effects of genetics and environment on the etiology of internalizing outcomes in sexual and gender minorities.
